# Probing nanoscale spatial distribution of plasmonically excited hot carriers

**DOI:** 10.1038/s41467-020-18016-4

**Published:** 2020-08-24

**Authors:** Sheng-Chao Huang, Xiang Wang, Qing-Qing Zhao, Jin-Feng Zhu, Cha-Wei Li, Yu-Han He, Shu Hu, Matthew M. Sartin, Sen Yan, Bin Ren

**Affiliations:** 1 State Key Laboratory of Physical Chemistry of Solid Surfaces, Collaborative Innovation Center of Chemistry for Energy Materials (iChEM), College of Chemistry and Chemical Engineering, Xiamen University, Xiamen, 361005 China; 2 Institute of Electromagnetics and Acoustics, Xiamen University, Xiamen, 361005 China

**Keywords:** Photochemistry, Photochemistry, Nanoscience and technology, Nanoscience and technology

## Abstract

Surface plasmons (SPs) of metals enable the tight focusing and strong absorption of light to realize an efficient utilization of photons at nanoscale. In particular, the SP-generated hot carriers have emerged as a promising way to efficiently drive photochemical and photoelectric processes under moderate conditions. In situ measuring of the transport process and spatial distribution of hot carriers in real space is crucial to efficiently capture the hot carriers. Here, we use electrochemical tip-enhanced Raman spectroscopy (EC-TERS) to in situ monitor an SP-driven decarboxylation and resolve the spatial distribution of hot carriers with a nanometer spatial resolution. The transport distance of about 20 nm for the reactive hot carriers is obtained from the TERS imaging result. The hot carriers with a higher energy have a shorter transport distance. These conclusions can be guides for the design and arrangement of reactants and devices to efficiently make use of plasmonic hot carriers.

## Abstract (web summary)

Revealing the spatial distribution of hot carriers in real space is crucial to their efficient utilization. Here, the authors show that in-situ electrochemical tip-enhanced Raman spectroscopy is able to resolve the spatial distribution of reactive hot carriers with a nanometer spatial resolution.

## Introduction

Surface plasmons (SPs) of metal nanostructures are able to dramatically improve the absorption of light and confine the optical electric field to a nanoscale region. The nonradiative decay of SPs via Landau damping creates hot carriers with an energy significantly higher than that can be achieved by the thermal excitation, even when the system is kept at ambient temperature^1–7^. These hot carriers can be transferred to semiconductors and molecules on the surface, driving the chemical processes. As a result, SPs provide a highly efficient way to manipulate light at the nanoscale to realize the efficient transformation of substances and energy for photocatalysis^1–7^ and photovoltaics^1,2,8^.

After generation in the nanoscale localized and strongly enhanced plasmonic electric field, the hot carriers can only travel a few tens of nanometers before they lose their energy by thermalization via the electron–electron scattering and electron–phonon scattering, which has been revealed by the theoretical calculation^9,10^. The consequent spatial distribution of the hot carriers may determine the active area on the surface, which is crucial to efficiently capture the hot carriers for achieving highly efficient SP-based catalysts or optoelectronic devices^5,9,11^. Recently, the spatial distribution of hot carriers has been visualized via ex situ scanning electron microscope or florescence imaging of the reaction or stained products of the hot carriers-induced reaction^11–14^. It is still desired and remains challenging to in situ measure the transport process and spatial distribution of hot carriers in real space, which requires not only a flexible control of the SP-catalyzed reaction but also a characterization technique with a nanometer spatial resolution to resolve the distribution of the reaction in real space.

Electrochemical tip-enhanced Raman spectroscopy (EC-TERS)^15–20^ is a nanospectroscopic technique, which integrates ultrahigh spatial resolution^21,22^ and rich vibrational (fingerprint) information of TERS and flexible control of the energy of the electrode materials of electrochemistry. The SP effect in the coupled plasmonic tip and substrate gap could not only amplify the Raman scattering of the species in the gap but also drive the SP-catalyzed reaction simultaneously^23–25^. Therefore, EC-TERS can be used to in situ characterize the SP-driven chemical reaction.

Here, we use EC-TERS to in situ monitor a SP-driven decarboxylation reaction at the nanoscale and resolve the spatial distribution of reactive hot carriers beneath the plasmonic tip by fully exerting its unique advantages. We tune the Fermi level of the electrode by changing the applied potential to turn on and off (during TERS mapping) the SP-driven reaction. The spatial distribution of reactive hot carriers is visualized in real space by EC-TERS, from which the transport distance of about 20 nm for the reactive hot holes can be obtained. Further, we demonstrate in experiment that the hot holes with a higher energy have a shorter transport distance. These conclusions can be guides for the spatial arrangement of the reactant molecules and devices to efficiently make use of the hot carriers in the design of the SP-based catalysts.

## Results

### In situ monitoring the SP-driven decarboxylation by EC-TERS

**Fig. 1:**
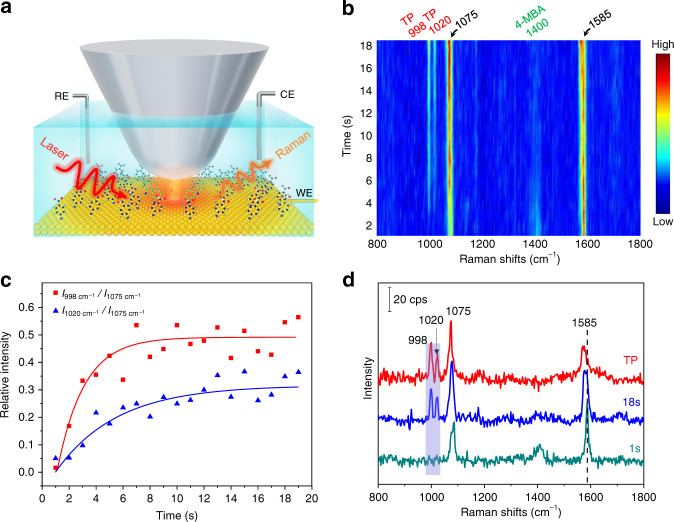
Monitoring the decarboxylation of 4-MBA on Au(111). **a** Schematic illustration of the EC-TERS setup, where the decarboxylation reaction is induced by the strongly coupled SP between the Ag tip and Au substrate. **b** Time series TERS spectra of 4-MBA in 0.1 M NaClO_4_ (pH 10). Power: 1.1 mW, bias: 200 mV, *I*_tunneling_: 800 pA, and *E*_sample_: −0.32 V. **c** Plots of the relative intensities of the bands at 998 and 1020 cm^−1^ (assigned to TP) to that at 1075 cm^−1^ (combined contribution of TP and 4-MBA) against time. **d** TERS spectra of 4-MBA (at 1 and 18 s) and TP.

For this study, we chose the decarboxylation reaction, a key step in Kolbe electrolysis reaction^26^, as the model reaction for the EC-TERS study (Fig. 1a). 4-mercaptobenzoic acid (4-MBA) molecules, which have no absorption at the wavelength of the excitation laser, were used as reactants. They were anchored on Au(111) surface via the strong Au–S bond, so that they formed a dense, self-assembled monolayer (Supplementary Fig. 1). Anchoring the molecules to the electrode in this way reduces the influence of molecular diffusion on the surface and improves the reliability of imaging. At open circuit potential (~−0.32 V), the time series TERS spectra (Fig. 1b) show that the intensity of Raman peak at 1400 cm^−1^ assigned to COO^−^ stretching vibration (Supplementary Table 1) decreases over time, accompanied by the appearance and growth of two new peaks at 998 and 1020 cm^−1^. The peak intensities reach constant values after several seconds (Fig. 1c). To analyze the spectral changes, we compare in Fig. 1d the first and last spectra of the time series. The two new peaks show similar frequency and relative peak intensity to that of thiophenol (TP) (Fig. 1d red spectrum and Supplementary Table 2), indicating that 4-MBA molecules have been decarboxylated to form TP through plasmonic photocatalysis^27,28^. Control experiments show that the reaction could only be initiated with the participation of both the Ag tip and photon (Supplementary Note 1, Supplementary Figs. 4, 5), demonstrating that the decarboxylation reaction was driven by the assistance of the SP. In electrochemistry, inducing a decarboxylation process requires a high overpotential^26^, whereas it can now occur even at open circuit potential with the assistance of the SP^27,28^.

### Mechanism and modulation of the SP-driven reaction

**Fig. 2:**
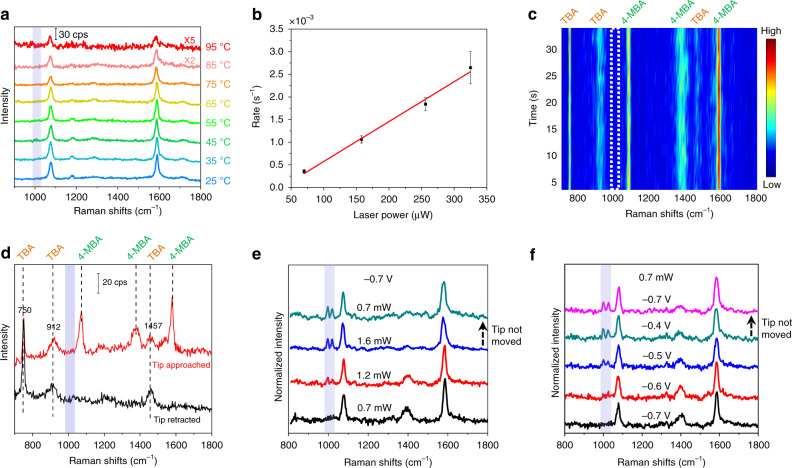
Study of the catalytic mechanism and reaction modulation. **a** Ex situ TERS spectra of 4-MBA after the system temperature was gradually heated to 95 °C. Bias: 200 mV, *I*_tunneling_: 600 pA. **b** Power-dependent decarboxylation reaction rate measured by SERS on a substrate consisting of plasmonic Au NPs in 0.1 M NaClO_4_ (pH 10). The error bars represent the standard deviations (s.d.) for three measurements. **c** Time series TERS spectra of 4-MBA in NaClO_4_ solution (pH 10) contained 2.7 M TBA. **d** Representative spectra when the tip was approached to and retracted from the 4-MBA adsorbed Au(111) surface in NaClO_4_ solution (pH 10) containing 2.7 M TBA. The peaks at 750, 912, and 1457 cm^‒1^ are the Raman peaks of TBA in the solution. Laser power: 1.1 mW. Bias: 200 mV, *I*_tunneling_: 800 pA, *E*_sample_: ‒0.32 V. **e** Power-dependent and **f** potential-dependent EC-TERS spectra. The potentials of substrate are referred to the Pt quasi reference electrode. At each power or potential, the tip was moved to a new position on the Au(111) substrate. After the tip was moved to a new position and the sample was illuminated by the laser for 30 s, the TERS spectra were acquired with an integration time of 5 s. The intensity is normalized by the 1075 cm^‒1^ peak. The sample potential and laser power are included in the figures. Bias: 100 mV, *I*_tunneling_: 800 pA.

The photocatalytic reaction can be facilitated by hot carriers or the photothermal effect resulting from the SP relaxation^6,29^. Recently, it was reported that the hot carrier was responsible for the SP-driven decarboxylation^28^. To further rule out that the decarboxylation process of 4-MBA was induced by a thermal effect, we performed two control experiments. First, we heated the 4-MBA-adsorbed Au(111) electrode in a temperature-controlled cell containing 0.1 M NaClO_4_ (pH 10) solution, then moved the electrode and measured the TERS spectra in air at room temperature. As shown in Fig. 2a, the feature peaks of TP are absent when the system is heated from 25 to 75 °C. When the temperature is further increased to 85 and 95 °C, the TERS intensity of 4-MBA decreases, due to thermal desorption of molecules. The feature peaks of TP are still absent even at the desorption temperature, indicating that the decarboxylation of the molecules adsorbed on surface might not be induced by heating.

Second, we performed in situ SERS experiments to study the role of heat. In our previous work, we have demonstrated that the Raman shift of the N≡C stretching peak is sensitive to the surface temperature, which can be used as a nanoscale temperature indicator in SERS^30^ (Supplementary Fig. 6a). With this method, we measured the surface temperature (about 45 °C) of the Au NPs using the laser power that could induce the reaction by SP excitation (Supplementary Note 2 and Supplementary Figs. 6, 7), then the system was heated to the same temperature. The absence of the Raman features from the product TP (Supplementary Fig. 8) indicates that the thermal effect is not the dominant effect in the SP-induced decarboxylation of 4-MBA. This conclusion is further supported by the linear dependence of the reaction rate on the laser power, because the thermal effect would expect an exponential dependence predicted by the Arrhenius equation^6,29^ (Fig. 2b and see detailed information in Supplementary Note 3).

Decarboxylation is an anodic process in electrolysis^26^. In photocatalytic decarboxylation (Photo-Kolbe) reactions, photo-generated holes can induce decarboxylation following two possible pathways^31^: (i) holes react with carboxylate and induce decarboxylation; (ii) holes oxidize OH^−^ or H_2_O to produce the hydroxyl radical OH·, then the OH· initiates decarboxylation of carboxylate.

1 $${\mathrm{(i)}} \ {\mathrm{R}} - {\mathrm{COO}}^{-} + {\mathrm{h}}^{+} \to {\mathrm{R\cdot}} + {\mathrm{CO}}_{2}$$

2 $${\mathrm{(ii)}} \ {\mathrm{h}}^{+} + {\mathrm{OH}}^{-} \to {\mathrm{OH\cdot}}$$

3 $${\mathrm{R}} - {\mathrm{COO}}^{-} + {\mathrm{OH\cdot}} \to {\mathrm{R\cdot}} + {\mathrm{CO}}_{2} + {\mathrm{OH}}^{-}$$

The difference between the two pathways is whether there is OH· participating in the reaction. To examine the role of OH· in the SP-driven decarboxylation, Tert-butanol (TBA), which is a scavenger of OH· and frequently used for quenching OH·^32–34^, was added to the electrolyte solution. As shown in Fig. 2c, d, when TBA is present in the solution, the feature peaks of TP are absent in the TERS spectra, and the intensity of COO^−^ vibrational peak does not decrease over time under continuous laser illumination (similar SERS experiment results are shown in Supplementary Fig. 10). This result indicates that the reaction is quenched by the TBA. It demonstrates that the decarboxylation is initiated by OH·. We further performed experiment with terephthalic acid, which can react with OH· to form a fluorescing molecule of 2-hydroxyterephthalic acid^35–37^. Indeed, we observed a strong fluorescence peak at 425 nm after reaction, indicating the formation of OH· (Supplementary Note 5 and Supplementary Fig. 11). These controlled experiments also confirm that the chemical process is mainly induced by hot carriers rather than the plasmonic thermal effect.

Hot carriers are generated from the photoexcitation of electrons below the Fermi level. In electrochemistry, the Fermi level of the electrode can be precisely controlled by changing the electrode potential. Thus, it is possible to regulate the energy and reactivity of plasmonic hot carriers by electrochemistry. To ensure the experimental reliability, the tip was moved to a new position on the Au(111) substrate for each power and potential condition. Figure 2e shows that the intensities of the peaks at 998 and 1020 cm^−1^ increase as the laser power increases from 0.7 to 1.6 mW, due to the larger number of hot carriers derived from photons at high laser power. In addition, the feature peaks of TP remain unchanged when the laser power is returned to 0.7 mW (the tip was not moved), indicating that the reaction is irreversible. To study the effect of potential on the decarboxylation reaction, we fixed the laser power at a low value (0.7 mW) and varied the potential. At −0.7 V, the feature peaks of TP are absent. As the potential is gradually shifted toward −0.4 V, the intensities of the feature peaks of TP increase (Fig. 2f), indicating that the reaction is promoted by the positive shift of potential. Because the decarboxylation reaction is irreversible, the feature peaks of TP also remained when the potential was switched to −0.7 V. It is interesting to find that a positive shift of the electrode potential by only 0.3 V can achieve an improvement in the reactivity equivalent to an increase of laser power by 0.9 mW. Therefore, the reaction can now be triggered at a positive potential with a low laser power, avoiding the photodamage induced by the high laser power.

**Fig. 3:**
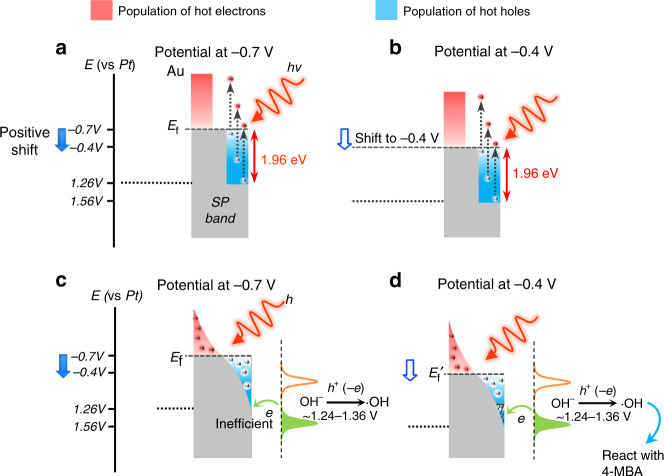
Effect of potential on the carrier energy and the reaction. Panels **a** and **b** illustrate the excitation of the hot carriers, **c** and **d** illustrate the population of carriers after decaying to follow the Fermi–Dirac-like distribution. Panels **a** and **c** are the energy distributions of carriers at ‒0.7 V, **b** and **d** are the energy distributions of carriers at ‒0.4 V. (All potentials are referenced to a Pt electrode. The energy axis is not to scale for clarity).

The modulation process of the SP-driven reaction with applied potentials could be interpreted and analyzed via a simple energy level diagram (Fig. 3). In the TERS configuration, the strongly coupled SP between the Ag tip and Au substrate is excited by 632.8 nm (1.96 eV) laser light. The SP could be damped through the creation of hot electron–hole via Landau damping. The scattering of electrons by surface or in the highly confined electromagnetic fields in hot spot enables the nonconservation of the linear momentum of electrons. The electrons can absorb the full energy of the photon to generate the high-energy hot carriers^38,39^. Therefore, the electrons that reside below the Fermi level are excited to a higher, unoccupied energy level, generating hot electrons (between *E*_f_ and *E*_f_ +1.96 eV) and leaving holes with energies between *E*_f_ −1.96 eV and *E*_f_ (Fig. 3a, b). The hot carriers then quickly redistribute their energy via electron–electron scattering processes following a time-dependent Fermi–Dirac-like distribution^2^ (Fig. 3c, d). The energy distribution of the hot carriers determines their catalytic ability and efficiency. The carriers with energies more positive than the oxidization potential of OH^−^ are considered as reactive hot carriers (in the present system, reactive hot holes). At pH 10, the potential of the redox OH·/OH^−^ is 1.77 – 1.89 V vs NHE^40,41^, which is about 1.24 – 1.36 V vs a Pt quasi reference electrode. At −0.7 V, the maximal energy (most positive potential) of SP-generated hot holes with 632.8 nm excitation is 1.26 V (Fig. 3a). However, the number of reactive hot holes is extremely low (Fig. 3c). As a result, the probability of inducing the reaction is low under these conditions. When the potential is shifted positively to −0.4 V, the maximal energy of hot holes is shifted up to 1.56 V (Fig. 3b). Under this condition, there is a much greater number of holes with sufficient energy to oxidize OH^−^ than that at −0.7 V (Fig. 3d), leading to a dramatically increased reaction rate. Here the SP-induced reaction was tuned by modulating the energy of hot holes with the change of the potential while fixing the illumination wavelength. The reaction can also be tuned by changing the illumination wavelength^42^. Both approaches can be understood within a simplified and unified scenario shown in Supplementary Fig. 12.

In the present study, the hot carriers could be generated both in the Au substrate and Ag tip in the plasmonic nanocavity. The tip potential- and substrate potential-dependent experiment reveals that the reaction is mainly triggered by the hot carriers generated in the Au substrate (Supplementary Note 6 and Supplementary Fig. 13). The generated hot carriers could offer the energy to overcome the reaction barrier, leading to the reduction of required potential. Compared with uncoupled single nanoparticles^42,43^, the coupled plasmonic nanocavity with only about 1 nm gap between the Ag tip and Au substrate here can generate a more intense and confined electric field in the hot spot. Such a highly intense and confined field facilitates the breaking of linear momentum-matching conditions^44,45^, which improves the efficiency to generate high-energy carriers to overcome the reaction barrier and leads to an effective reduction in the potential. The potential of the hot holes could be more positive than that for the Au oxidation or sulfydryl oxidation. However, we did not observe the peak attributed to the oxidation product, owing to the low reaction efficiency at the controlled potential together with the small Raman scattering cross sections of the species (Supplementary Note 7). Here one may argue that the variation of the reactivity with potential could be attributed to the shift of SP resonance at different potentials, which may lead to different SP resonances at the laser wavelength. We found that the backgrounds of the TERS spectra do not shift with the change of the potential (Supplementary Fig. 14), indicating that the potential did not lead to an obvious change in the SP resonance response. It demonstrates that the change in the reactivity at different potentials is not due to the shift of SP resonance.

### Spatial profile of the reactive hot carriers

**Fig. 4:**
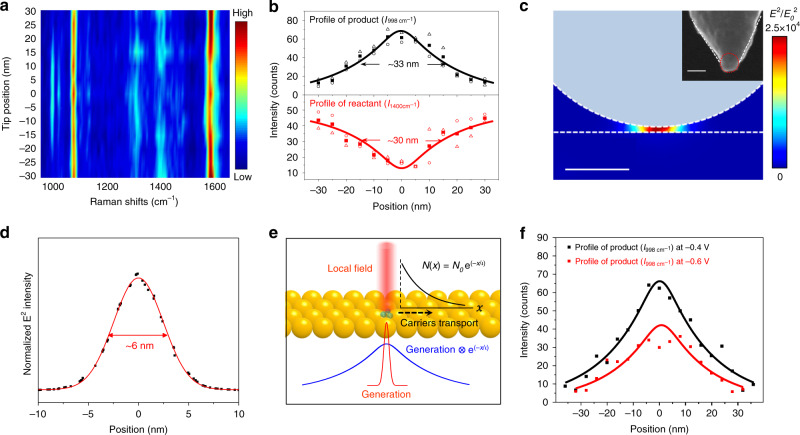
Profile of the reaction region on the substrate under Ag tip. **a** Color-coded intensity map of the line-trace TERS spectra across a reaction region. The reaction was induced at the sample potential of −0.4 V under a laser power of 0.7 mW. The line-trace TERS spectra were acquired with the sample potential at −0.7 V. Bias: 100 mV, *I*_tunneling_: 800 pA. **b** Plots of intensities of the 998 cm^−1^ peak (top) and 1400 cm^−1^ peak (bottom) with the tip position. The open triangle, circle symbols represent the intensities in the trace and retrace spectra. The solid square symbols represent the average intensities of the trace and retrace spectra. The color-coded intensity map and the Raman spectra of the trace and retrace are shown in Supplementary Fig. 16. The fitted lines were obtained by using the function $AE(x)^{\mathrm{2}} \otimes {\mathrm{e}}^{({ - | x |/{\mathrm{\iota }}})} \otimes R(x)$, where *A* is a constant, $E( x )^{\mathrm{2}}$ is the profile of plasmonic electric field intensity in **d**, *R*($x$) is the TERS spatial resolution profile obtained from Supplementary Fig. 15. **c** Calculated plasmonic electric field (*E*^2^) distribution when the Ag tip is positioned on the Au surface, the scale bar is 10 nm. Inset: scanning electron microscope image of the Ag tip, the scale bar is 50 nm. **d** Calculated *E*^2^ profile on the substrate surface in the plasmonic gap. Red line is fitted using a Gaussian function. **e** Schematic illustration of hot carrier generation and transport on surface. **f** Profile of the reaction induced at −0.6 and −0.4 V for comparison. The color-coded intensity maps of the line-trace TERS spectra are shown in Supplementary Fig. 19. Bias: 100 mV, *I*_tunneling_: 800 pA.

We have verified that the reaction is driven by hot holes generated by the strongly coupled and localized SP in the gap between the tip and substrate. The reaction can be triggered and the spectral information during the chemical processes can be recorded simultaneously with TERS. Furthermore, the spatially resolved molecular spectra, obtained by TERS may give us an opportunity to reveal the distribution of reactive hot holes in real space. However, since the tip plays the roles of both reaction initiator and signal amplifier, the reaction may be continuously triggered following the tip trace in conventional TERS, which makes it a great challenge to precisely image the reaction profile in the SP gap between the plasmonic tip and substrate. Here, we take advantage of EC-TERS, which allows flexible control of the reaction by controlling the potential. With the tip held at a fixed position, the substrate potential was first set to −0.4 V to induce the reaction. Once the peak intensities of the 998 and 1020 cm^−1^ bands reached constant values, the potential was switched to −0.7 V to stop the reaction. In this way, the spatial profile of the TP that had been generated at the single position could be preserved and mapped using TERS without perturbing the reaction further. As shown in Fig. 4a, b, when the tip is scanned from the perimeter of the reaction region toward its center, the peak intensities of the product at 998 and 1020 cm^−1^ gradually increase and reach maximum at the center, while the peak intensity of the reactant at 1400 cm^−1^ shows the opposite trend. The symmetrical and correlated distributions of the reactant and product show that the FWHM of the reaction profile is about 30 nm, which is similar to that observed with in situ UHV-STM of a plasmon-induced reaction^46^ but much larger than the 5 nm spatial resolution of the TERS system (Supplementary Note 8 and Supplementary Fig. 15). Therefore, the spatial profiles of the product and reactant are not artifacts limited by the spatial resolution of the instrument. Furthermore, the 30 nm reaction profile is also much larger than that of the local field distribution of about 6 nm (as will be discussed later in Fig. 4c, d). Therefore, the near field driven direct energy transfer between molecules and metal^47^ may not be the main mechanism for the reaction.

Considering the reaction is initiated by OH· generated by reactive hot holes, the size of the reaction area may be determined by the spatial distribution of the reactive hot holes and the diffusion length of OH·. However, the diffusion length of OH· is estimated to be only about 1.7 nm (Supplementary Note 9), which is far smaller than the range of the reaction size. Therefore, the reactive area in the coupled plasmonic gap is mainly determined by the distribution of reactive hot holes, which depends on both the initial generation and transport of the carriers^5,11^. Since the generation probability of hot carriers (*G*($x$)) is proportional to the intensity of plasmonic electric field (${E}\left( x \right)^{2}$) inside the metal, that is, $G(x)$ ∝ ${{E}}\left( x \right)^2$, the profile of the initial generated hot carriers will follow the profile of the localized plasmonic electric field. Theoretical simulation of the Ag tip using the geometry parameters in the experiment were carried out to calculate the distribution of local fields in the plasmonic gap between the tip and substrate (Fig. 4c, d). As the intensity of the electric field decreases dramatically inside the metal, we use the local field distribution on the top surface of the substrate as the maximum of the one inside the metal. The FWHM of the local field distribution is about 6 nm (Fig. 4d), which is much smaller than the size of the reaction region. From the above rationing, carrier transport processes may play an important role in determining the final spatial distribution of hot carriers. Here, the well-defined and atomically smooth Au(111) crystal was used as the substrate. Thus, scattering by defects and impurities within the metal during the transport of hot carriers could be avoided. It has been reported that an exponential attenuation model $N\left( x \right) = N_0{\mathrm{e}}^{\left( { - x/\iota } \right)}$ could be used to estimate the number of hot carriers with distance^48,49^. Here, *ι* is the decay length of hot carriers, $N_0$ and $N\left( x \right)$ are the number of the hot carriers at the generated site and after transport for a distance of $x$, respectively. Taking the transport of carriers into consideration, the spatial distribution of hot carriers is a convolution of the generation and transport in real space (Supplementary Note 10, Supplementary Fig. 17 and Fig. 4e), that is, the final spatial distribution of hot carriers $D\left( x \right) = {{G}}\left( x \right) \otimes {\mathrm{e}}^{\left( { - \left| x \right|{\mathrm{/\iota }}} \right)} \propto {{E}}\left( x \right)^{\mathrm{2}} \otimes {\mathrm{e}}^{\left( { - \left| x \right|{\mathrm{/\iota }}} \right)}$. The experimental imaging result is a convolution of the final spatial distribution of hot carriers and the TERS spatial resolution, so that the reaction profile obtained from the TERS line trace can be expressed as ${P {({x})}} = {{{D}}} {{({x})}} \otimes {{{R}}} {({x})} \propto {{{E}}} {{({x})}^{2}} \otimes {\mathrm{e}^{(-|{x}|/{\iota})}} \otimes {{{R}}} ({x})$. *R*($x$) is the TERS spatial resolution profile. Using this convolution model to fit the profile of the product and reactant in Fig. 4b, the transport distance (*ι*) of the reactive hot holes was estimated to be around 20 nm for the reactive hot holes that could overcome the energy barrier of about 1.66 eV for oxidation reaction. If a reaction has a different reaction barrier that requires carriers with more / less energies to overcome, the transport distance of the reactive hot carriers may be different. In the following, we discuss the variation of spatial profile of reactive hot carriers with the energy barrier. To the best of our knowledge, quantitatively extracting the spatial range of reactive plasmonic hot carriers in real space has not been achieved before.

### Potential effect on the spatial profile of reactive carriers

In EC-TERS, we can conveniently tune the energy of hot carriers by changing the potential of the substrate. In this way, the transport distance can also be tuned. When we shifted the potential from −0.4 to −0.6 V, we increased the energy barrier for OH^−^ oxidation from about 1.66 to 1.86 eV (relative to the Fermi level) and reduced the reactivity and the reaction region (Fig. 4f). The transport distance estimated using the data in Fig. 4f is ~17 nm at −0.6 V and ~20 nm at −0.4 V for the hot holes with energies higher than 1.86 and 1.66 eV, respectively, relative to the Fermi level. Such a difference can be convincingly resolved in our TERS system as the measurement error was only about 1.1 nm (Supplementary Fig. 18). It indicates that the hot holes with higher energy could only be collected within a shorter distance, agreeing with the reported simulation result due to the shorter mean free path of hotter carriers^9,11^. At −0.6 V, the energy barrier is only about 0.1 eV lower than the excitation laser energy (1.96 eV). Therefore, most of the reactive hot carriers will not experience multiple scattering events^50^. As a result, the transport distance is comparable to the reported mean free path of the hot carriers in Au^10^.

## Discussion

In summary, we visualized the nanoscale reaction region beneath the tip with EC-TERS imaging of a plasmon-driven decarboxylation reaction by turning on and off the reaction with potential control. We obtained the transport distance for the reactive hot holes in real space, which is comparable to the mean free path of the hot carriers in Au. We demonstrated in experiment that the hot holes with a higher energy have a shorter transport distance. The conclusions obtained in this study may guide the design of efficient photocatalysis and optoelectronics by placing the reactant molecules or charge collectors within the transport distance of reactive hot carriers to efficiently collect and utilize the hot carriers.

## Methods

### Preparation of Au(111) and 4-MBA self-assembled monolayers

The Au(111) single crystal bead was fabricated by Clavilier’s method^51^ and was electrochemically polished and flame-annealed before use. After treatment, the Au(111) single crystal was immersed in a 1 mM ethanolic solution of 4-mercaptobenzoic acid (4-MBA) for 120 min to allow the formation of a self-assembled monolayer (SAM) on the surface. The sample was rinsed with ethanol and dried under N_2_ atmosphere. Then it was used for TERS and EC-TERS studies.

### EC-TERS setup and EC-TERS experiment

The EC-TERS setup used in the current study combined an EC-STM (Nanoscope E, Veeco, USA) with a home-built Raman microscope via a side-illumination configuration^20^. The laser light was brought in from the left side of the cell at a tilted angle of 70° relative to the tip axis (Supplementary Fig. 2). To avoid distortion of the optical path as a result of the refractive index mismatch in an electrochemical system and to increase the excitation and collection efficiency, a water immersion objective with a high numerical aperture (NA) of 0.8 was used to focus the laser beam and collect TERS signals. On considering that the working distance of the objective was only 3.2 mm, we modified the STM scanner to gain more open space for accommodating the microscope objective. The objective was installed on a three-dimensional piezo stage so that the objective could be smoothly adjusted to precisely focus the laser spot onto the plasmonic tip apex without introducing mechanical shock.

A home-built EC cell^20^ was used for EC-TERS (Supplementary Fig. 2b). The EC-TERS cell was a Kel-F (poly(chlorotrifluoro-ethylene)) body with a hole in the bottom to install the homemade Au(111) working electrode. A flat thin glass window was attached to the left side and tilted part of the cell for the transmission of laser and Raman signal. A droplet of water was added in the gap between the objective and the glass window to reduce the mismatch of the refractive index of the media in the optical path. A platinized Pt wire was used as the counter electrode and a Pt wire was used as the quasi reference electrode. The two electrodes were fixed on the right side of the EC cell. 0.1 M NaClO_4_ solution was used as the supporting electrolyte to reduce the ohmic drop of solution. The pH of the electrolyte was adjusted to about 10 using NaOH solution. In EC-TERS experiments, an electrochemically etched Ag tip prepared according to the reported protocol^52,53^ was used to obtain the TERS spectra. The tip was further coated with melted polyethylene glue (Rapid Pro^+^ glue, Sweden) to reduce the exposed tip length to ~1 μm^15^, so that the Faradaic current produced at the tip became so small that it would not interfere with the tunneling current. The potentials of the substrate and the tip were controlled by the built-in bipotentiostat of the EC-STM.

### TERS characterization of 4-MBA on Au(111) after heating in water bath

The 4-MBA-adsorbed Au(111) electrode in a glass cell containing 0.1 M NaClO_4_ (pH 10) solution was heated at the controlled temperature, which was maintained at the temperature for at least 15 min. The electrode was then moved from the solution and characterized with TERS in air and at room temperature. Note that the decarboxylation is an irreversible reaction and could not be induced by the SP in air during the TERS characterization. Therefore, we could safely use the ex situ characterization to examine if the molecules on surface have been decarboxylated at the desired temperature.

### TERS line-trace imaging measurement

To obtain a reaction profile induced by surface plasmon (SP) in the tip-substrate gap, the substrate potential was first set at a potential more positive than −0.7 V, e.g., −0.4 or −0.6 V, to induce the reaction. Once the peak intensities of the 998 and 1020 cm^−1^ bands reached constant values, the potential was switched to −0.7 V, where the reaction could not occur, to obtain a TERS line trace without inducing additional reactions. In the TERS line-trace imaging experiment, the laser power used was 0.7 mW, the tip was scanned at a rate of 1 nm s^−1^, and TERS spectra were acquired simultaneously with an integration time of 4 s in Fig. 4f or 5 s in Fig. 4a, b. With these parameters, the step size of TERS imaging was estimated to be 4 or 5 nm.

### SERS experiments

The SERS experiments were performed on a substrate assembled with Au NPs (with a diameter of 55 nm)^30,54^. The substrate was immersed in an ethanolic solution of 1 mM 4-MBA for 120 min, and cleaned with copious amount of ethanol. Then, it was put in a glass cell containing 0.1 M NaClO_4_ (pH 10) solution. The spectra were acquired on a confocal Raman instrument (Alpha 3000, WITec, Germany). A water immersion objective (Nikon, ×40, NA 0.8) was used for exciting and collecting the Raman signal.

### Electromagnetic field simulation

Theoretical simulation with a model based on the geometry parameters of the tip used in the experiment were carried out to calculate the distribution of local fields in the strongly coupled and localized plasmonic gap between the Ag tip and Au substrate. The scanning electron microscope image of the Ag tip shows a tip radius of 25 nm and the cone angle of 50°. COMSOL Multiphysics 5.2a based on the finite element method was used for the computational simulations. The *p*-polarized light with an incidence angle of 20° was introduced by a user-defined port (the blue part in Supplementary Fig. 3). The perfect matched layer (PML) boundary conditions were adopted for all other outer interfaces. The refractive index of water was set to 1.33. Optical constants for Au and Ag were obtained from the literature^55,56^. All materials were assumed to be isotropic and nonmagnetic in the simulation. Extremely fine meshing with edge length smaller than the skin depth in the metal material was adopted to discretize the structure and to ensure convergence and reproducibility of the simulations. The minimum edge length of the tetrahedral mesh element was as small as 0.2 nm.

## Supplementary information

Supplementary Information

## Data Availability

The data that support the findings of this study are available from the corresponding author upon reasonable request.

## Code availability

The code used for convolution fitting in this study have been archived in a GitHub repository (https://github.com/99623/convolution-fitting-code/releases/tag/1.0).

## References

[1] Clavero C (2014). Plasmon-induced hot-electron generation at nanoparticle/metal-oxide interfaces for photovoltaic and photocatalytic devices. Nat. Photonics.

[2] Brongersma ML, Halas NJ, Nordlander P (2015). Plasmon-induced hot carrier science and technology. Nat. Nanotechnol..

[3] Linic S, Aslam U, Boerigter C, Morabito M (2015). Photochemical transformations on plasmonic metal nanoparticles. Nat. Mater..

[4] Christopher P, Moskovits M (2017). Hot charge carrier transmission from plasmonic nanostructures. Annu. Rev. Phys. Chem..

[5] Narang P, Sundararaman R, Atwater HA (2016). Plasmonic hot carrier dynamics in solid-state and chemical systems for energy conversion. Nanophotonics.

[6] Kale MJ, Avanesian T, Christopher P (2014). Direct photocatalysis by plasmonic nanostructures. ACS Catal..

[7] Zhan C (2018). From plasmon-enhanced molecular spectroscopy to plasmon-mediated chemical reactions. Nat. Rev. Chem..

[8] Atwater HA, Polman A (2010). Plasmonics for improved photovoltaic devices. Nat. Mater..

[9] Jermyn AS (2019). Transport of hot carriers in plasmonic nanostructures. Phys. Rev. Mater..

[10] Brown AM, Sundararaman R, Narang P, Goddard WA, Atwater HA (2016). Nonradiative plasmon decay and hot carrier dynamics: effects of phonons, surfaces, and geometry. ACS Nano.

[11] Cortés E (2017). Plasmonic hot electron transport drives nano-localized chemistry. Nat. Commun..

[12] Lee SJ, Piorek BD, Meinhart CD, Moskovits M (2010). Photoreduction at a distance: facile, nonlocal photoreduction of Ag ions in solution by plasmon-mediated photoemitted electrons. Nano Lett..

[13] Kim NH, Meinhart CD, Moskovits M (2016). Plasmon-mediated reduction of aqueous platinum ions: the competing roles of field enhancement and hot charge carriers. J. Phys. Chem. C..

[14] Simoncelli S, Li Y, Cortés E, Maier SA (2018). Nanoscale control of molecular self-assembly induced by plasmonic hot-electron dynamics. ACS Nano.

[15] Zeng Z-C (2015). Electrochemical tip-enhanced Raman spectroscopy. J. Am. Chem. Soc..

[16] Kurouski D, Mattei M, Van Duyne RP (2015). Probing redox reactions at the nanoscale with electrochemical tip-enhanced Raman spectroscopy. Nano Lett..

[17] Mattei M (2017). Tip-enhanced Raman voltammetry: coverage dependence and quantitative modeling. Nano Lett..

[18] Martín Sabanés N, Ohto T, Andrienko D, Nagata Y, Domke KF (2017). Electrochemical TERS elucidates potential-induced molecular reorientation of adenine/Au(111). Angew. Chem. Int. Ed..

[19] Yokota Y (2019). Systematic assessment of benzenethiol self-assembled monolayers on Au(111) as a standard sample for electrochemical tip-enhanced Raman spectroscopy. J. Phys. Chem. C..

[20] Huang S-C (2019). Electrochemical tip-enhanced Raman spectroscopy with improved sensitivity enabled by a water immersion objective. Anal. Chem..

[21] Zhang R (2013). Chemical mapping of a single molecule by plasmon-enhanced Raman scattering. Nature.

[22] Lee J, Crampton KT, Tallarida N, Apkarian VA (2019). Visualizing vibrational normal modes of a single molecule with atomically confined light. Nature.

[23] van Schrojenstein Lantman EM, Deckert-Gaudig T, Mank AJG, Deckert V, Weckhuysen BM (2012). Catalytic processes monitored at the nanoscale with tip-enhanced Raman spectroscopy. Nat. Nanotechnol..

[24] Sun M, Zhang Z, Zheng H, Xu H (2012). In-situ plasmon-driven chemical reactions revealed by high vacuum tip-enhanced Raman spectroscopy. Sci. Rep..

[25] Kumar N, Stephanidis B, Zenobi R, Wain AJ, Roy D (2015). Nanoscale mapping of catalytic activity using tip-enhanced Raman spectroscopy. Nanoscale.

[26] Schäfer H-J (1990). Recent contributions of kolbe electrolysis to organic synthesis. Top. Curr. Chem..

[27] Zong Y (2014). Plasmon-induced decarboxylation of mercaptobenzoic acid on nanoparticle film monitored by surface-enhanced Raman spectroscopy. RSC Adv..

[28] Huh H, Trinh HD, Lee D, Yoon S (2019). How does a plasmon-induced hot charge carrier break a C–C bond?. ACS Appl. Mater. Inter..

[29] Zhou L (2018). Quantifying hot carrier and thermal contributions in plasmonic photocatalysis. Science.

[30] Hu S (2018). Quantifying surface temperature of thermoplasmonic nanostructures. J. Am. Chem. Soc..

[31] Yoneyama H, Takao Y, Tamura H, Bard AJ (1983). Factors influencing product distribution in photocatalytic decomposition of aqueous acetic acid on platinized titania. J. Phys. Chem..

[32] Simic M, Neta P, Hayon E (1969). Pulse radiolysis study of alcohols in aqueous solution. J. Phys. Chem..

[33] Liu S, Zhang N, Tang Z-R, Xu Y-J (2012). Synthesis of one-dimensional CdS@TiO_2_ core–shell nanocomposites photocatalyst for selective redox: the dual role of TiO_2_ shell. ACS Appl. Mater. Inter..

[34] Fotiou T (2016). Assessment of the roles of reactive oxygen species in the UV and visible light photocatalytic degradation of cyanotoxins and water taste and odor compounds using C–TiO_2_. Water Res..

[35] Ishibashi K, Fujishima A, Watanabe T, Hashimoto K (2000). Quantum yields of active oxidative species formed on TiO_2_ photocatalyst. J. Photoch. Photobio. A.

[36] Chen X, Zhang J, Fu X, Antonietti M, Wang X (2009). Fe-g-C_3_N_4_-catalyzed oxidation of benzene to phenol using hydrogen peroxide and visible light. J. Am. Chem. Soc..

[37] Nosaka Y, Nosaka AY (2017). Generation and detection of reactive oxygen species in photocatalysis. Chem. Rev..

[38] Chang L (2019). Electronic structure of the plasmons in metal nanocrystals: fundamental limitations for the energy efficiency of hot electron generation. ACS Energy Lett..

[39] Hartland GV, Besteiro LV, Johns P, Govorov AO (2017). What’s so hot about electrons in metal nanoparticles?. ACS Energy Lett..

[40] Koppenol WH, Liebman JF (1984). The oxidizing nature of the hydroxyl radical. A comparison with the ferryl ion (FeO_2_^+^). J. Phys. Chem..

[41] Schwarz HA, Dodson RW (1984). Equilibrium between hydroxyl radicals and thallium (ii) and the oxidation potential of hydroxyl (aq). J. Phys. Chem..

[42] Pensa E (2019). Spectral screening of the energy of hot holes over a particle plasmon resonance. Nano Lett..

[43] Kim Y, Dumett Torres D, Jain PK (2016). Activation energies of plasmonic catalysts. Nano Lett..

[44] Sykes ME (2017). Enhanced generation and anisotropic Coulomb scattering of hot electrons in an ultra-broadband plasmonic nanopatch metasurface. Nat. Commun..

[45] Besteiro LV, Govorov AO (2016). Amplified generation of hot electrons and quantum surface effects in nanoparticle dimers with plasmonic hot spots. J. Phys. Chem. C..

[46] Kazuma E, Jung J, Ueba H, Trenary M, Kim Y (2018). Real-space and real-time observation of a plasmon-induced chemical reaction of a single molecule. Science.

[47] Seemala B (2019). Plasmon-mediated catalytic O_2_ dissociation on Ag nanostructures: hot electrons or near fields?. ACS Energy Lett..

[48] Soshea RW, Lucas RC (1965). Attenuation length of hot electrons in gold. Phys. Rev..

[49] Stollenwerk AJ (2008). Effect of interface band structure on hot-electron attenuation lengths in Au thin films. Phys. Rev. B.

[50] Ladstädter F, Hohenester U, Puschnig P, Ambrosch-Draxl C (2004). First-principles calculation of hot-electron scattering in metals. Phys. Rev. B.

[51] Clavilier J, Faure R, Guinet G, Durand R (1980). Preparation of monocrystalline Pt microelectrodes and electrochemical study of the plane surfaces cut in the direction of the {111} and {110} planes. J. Electroanal. Chem..

[52] Zhang W, Yeo BS, Schmid T, Zenobi R (2007). Single molecule tip-enhanced Raman spectroscopy with silver tips. J. Phys. Chem. C..

[53] Li M (2016). Electrochemical fabrication of silver tips for tip-enhanced Raman spectroscopy assisted by a machine vision system. J. Raman Spectrosc..

[54] Zheng X-S (2014). Laser power dependent surface-enhanced Raman spectroscopic study of 4-mercaptopyridine on uniform gold nanoparticle-assembled substrates. J. Phys. Chem. C..

[55] Palik, E. D. *Handbook of Optical Constants of Solids III* (Academic Press, Boston, 1998).

[56] Johnson PB, Christy RW (1972). Optical constants of the noble metals. Phys. Rev. B.

