# Supplementary material for: Probing nanoscale spatial distribution of plasmonically excited hot carriers
Source: Nat Commun. 2020 Aug 24;11:4211. doi: 10.1038/s41467-020-18016-4 (PMC7445266; doi:10.1038/s41467-020-18016-4)
Supplement: Supplementary file 1 — Supplementary Information [file 41467_2020_18016_MOESM1_ESM.pdf]

Supplementary information

Probing nanoscale spatial distribution of plasmonically excited hot carriers

Huang et al.

Supplementary Notes

Supplementary Note 1

Control experiments to examine the plasmon-assisted reaction.

We first examined whether the reaction could be simply induced by the tip in the absence of laser. The Ag tip was approached to 4-MBA-adsorbed Au(111) and fixed for 1 min, then the laser was illuminated on the tip to acquire the TERS spectra. As shown in Supplementary Figure 4, the feature peaks of TP are absent in the first spectrum, which demonstrates that approaching the tip alone in the absence of the laser illumination cannot induce the decarboxylation reaction. The spectral features of TP were observed only after laser illumination on the tip-substrate gap for several seconds. It demonstrates that laser illumination is required for the reaction, indicative of a photocatalytic reaction.

After that, we fixed the tip at one position and illuminated the tip until the observation of the reaction product. We then moved the tip to a new position but still within the laser spot to acquire the TERS spectra. Again, the first spectrum at the new position did not show the feature peaks of TP (Supplementary Figure 5), which indicates that the reaction could not be induced solely by laser illumination without the plasmonic tip. In other words, the reaction is a SP-induced process rather than a pure photocatalytic process.

Supplementary Note 2

The role of the plasmonic thermal effect demonstrated by heating experiments by in-situ SERS

In our previous work, we demonstrated that the Raman shift of the N≡C stretching peak is sensitive to the surface temperature, which can be used as a nanoscale temperature indicator in SERS (Supplementary Figure 6a).[1] We applied this method to measure the surface temperature of the Au NPs under a condition that could induce the reaction by the SP excitation, and then we heated the system to the same temperature to examine if the reaction was induced by the thermal effect. We assembled phenyl isocyanide (PIC) molecules on the substrate modified with gold nanoparticles, which was used to monitor the surface temperature under the laser illumination. We then obtained a working curve by performing temperature-dependent SERS measurements of the peak positions of the N≡C bond by heating the Au NPs-modified substrate in a temperature-controlled water bath.

We used a very low laser power (~8 μW) to measure the SERS spectra to avoid the laser heating effect. Supplementary Figures 6b and 6c show a linear shift of the peak position to a lower frequency with the increasing temperature. In parallel, we measured the laser-induced plasmonic heating effect by measuring the SERS spectra at different laser powers. As shown in Supplementary Figures 6d and 6e, the peak shifts to a lower frequency with the increasing laser power. By comparing the power-dependent and temperature-dependent peak positions, the temperature on the assembled Au NPs surface under the illumination of various laser powers can be obtained.

As shown in Supplementary Figure 7, the decarboxylation reaction of the 4-MBA on the plasmonic gold NPs could be induced when the laser power is higher than 158 μW. If we took the case of 325 μW laser power as an example, the surface temperature of Au NPs at this laser power was estimated to about 45 °C using the working curves shown in Supplementary Figures 6c and 6e. We then gradually heated the system in water bath to 45 °C and used SERS to monitor the process. Again, the laser power (~8 μW) for the SERS measurement was low enough to avoid any SP-induced reaction. As shown in Supplementary Figure 8, the feature peaks of TP were absent even after the system was heated to 45.8 °C, indicating that the plasmonic thermal effect alone was not the dominating mechanism in inducing the reaction.

Supplementary Note 3

SERS study of the laser power effect on the reaction rate

It has been pointed out in the literature that the reaction rates may depend differently on the laser power for the reactions dominated by the photothermal effect and the hot carrier effect.[2, 3] Therefore, investigation of the power-dependent reaction rate may provide information on the mechanism. For example, for the reactions mediated by the plasmonic heating, the reaction rate follows an exponential relationship with temperature, determined by Arrhenius expression. On considering the linear dependence of the surface temperature on the laser power,[4] an exponential dependence of the reaction rate on the laser power would be expected.[2] However, for reaction driven by the plasmonic hot carrier, both linear[5-7] and super linear[3, 8, 9] dependence of the reaction rate on the laser power have been observed. At a lower laser power, each reaction is induced by a single photon absorption event followed by the interactions of the resulting electronic charge carrier and the adsorbate. Therefore, we would expect to observe a linear dependence of the reaction rate on the laser power.

The superlinear dependence is only observed at a high laser power, when the molecule interacts with charge carriers derived from more than one photon, which is not the case for current study. Therefore, the laser power-dependent reaction rate may provide important evidence for determining the reaction mechanism. For this purpose, we plot the relative intensity of the bands at 998 cm^{-1} (contribution from TP) to that at 1075 cm^{-1} (combined contribution from both 4-MBA and TP) against time for various laser powers (Supplementary Figure 9). We could deduce the reaction rate from the slope of the initial linear part of curves. Indeed, the reaction rates display a linear dependence on the laser power, as shown in Supplementary Figure 9b, which confirms the SP driven decarboxylation reaction is a plasmonic hot carrier-induced process. One may argue that the Arrhenius expression for a plasmonic heating-mediated reaction may also exhibit a linear relationship over a sufficiently small range of laser powers. Therefore, the possibility of the plasmonic heating effect may not be completely ruled out by only showing the linear relationship. However, the combination of this linear dependence, the heating experiment (Figure 2a and Supplementary Figure 6-8), and the OH· radical scavenger experimental results (Figure 2c and Supplementary Figure 10), convincingly concludes that the plasmonic hot carrier is the dominant catalytic mechanism.

Supplementary Note 4

Difference of the Pt quasi reference electrode (Pt-QRE) potentials in the electrolyte with and without TBA

The difference of the Pt-QRE potentials in the $NaClO_4$ (pH 10) solutions with and without 2.7 M Tert-butanol (TBA) was about 0.03 V ($E_{Pt(without\ TBA)} - E_{Pt(with\ TBA)} \approx 0.03$ V). It means that the potential negatively shifts from –0.32 V to –0.35 V after addition of TBA, which is still much more positive than the minimum potential of –0.6 V required to induce the decarboxylation reaction (Figure 2f). We could conclude that the inhibition effect after addition of TBA is not due to the shift of the potential but due to the scavenger effect.

Supplementary Note 5

Detection of OH· by ex-situ fluorescence experiment

The OH· could be detected by using a fluorescent probe. Here, terephthalic acid (TA), a molecule

that can specifically react with OH· to form a fluorescent product[10-12], was used to confirm the presence of OH· during the SP-induced decarboxylation of 4-MBA. The 4-MBA molecules were adsorbed on the substrate fabricated with assembled plasmonic Au nanoparticles. Then the substrate was immersed in a 3 mM TA aqueous solution and illuminated by a focused 633 nm laser with a power of 0.33 mW. The laser was scanned over a region of about 4.5 μm × 4.5 μm to avoid the photo decomposition of the 4-MBA molecules. After the substrate was illuminated for 3.5 hours, the Raman signal of the substrate was measured and the solution was collected for fluorescence detection. Supplementary Figure 11a shows the typical spectra inside and outside the illuminated region. The presence of two new peaks at 998 cm^{-1} and 1020 cm^{-1} demonstrates that the region illuminated by 633 nm laser experienced a decarboxylation process. The reaction region can be clearly seen by the SERS image using the intensity ratio of 998 cm^{-1} to 1075 cm^{-1} peaks, shown in Supplementary Figure 11b. Meanwhile, the fluorescent peak at around 425 nm in the black curve in Supplementary Figure 11c measured from the collected solution originates from the emission of 2-hydroxyterephthalic acid, which is the reaction product between OH· and TA. The strong peak observed after laser illumination indicates the generation of the OH·. As a control experiment, the substrate was also put in the solution without laser illumination and the solution was collected for fluorescence detection. The fluorescence was hardly observed without laser illumination.

Supplementary Note 6

Effect of tip potential on the decarboxylation reaction

In the plasmonic nanocavity, the hot carriers could be generated both in the Au substrate and Ag tip. Both of them may be responsible for the decarboxylation. As shown in Supplementary Figure 13, when the substrate potential was fixed at –0.7 V and the tip potential was changed from –0.8 V to –0.5 V, no change was observed in the spectra (i and ii in Supplementary Figure 13). The same behavior occurred in iii and iv in Supplementary Figure 13 demonstrates that the reaction is not sensitive to the tip potential. On the other hand, when the tip potential was fixed at –0.5 V and the substrate potential was changed from –0.7 V to –0.4 V (ii and iv in Supplementary Figure 13), the peaks (998 cm^{-1} and 1020 cm^{-1}) attributed to the product of TP appeared in the spectra indicating that the reaction is modulated by the substrate potential. Therefore, we could conclude that the reaction was mainly induced by the hot carriers generated in the Au substrate.

Supplementary Note 7

Possibilities of other reactions on the surface

The potential range in our work was from –0.2 V to 0.1 V (vs NHE), which was more negative than the oxidation potential of Au (around 1.0 V vs NHE). Under the laser illumination (633 nm, 1.96 eV), hot holes with energies higher than 1.76 V vs NHE could be generated following the decay of the surface plasmon, whose energy was more positive than the oxidation potential of Au. However, the Au oxidation product staying on the surface could be reversibly reduced by the plenty of electrons near the Fermi level (–0.2 V to 0.1 V vs NHE) inside the Au and maintained the Au surface at a reduced state. Therefore, the amount of the Au oxidation species would be extremely small. On the other hand, when the OH^- diffuses to the Au surface, it is possible to be oxidized by the hot holes to form OH·. OH· has a weak interaction with the Au surface and can easily diffuse away from the surface, avoiding the complete reduction of OH· by the electrons located at the Fermi level. The remained OH· in the solution can induce the irreversible decarboxylation reaction of 4-MBA. Therefore, once the C-C is broken, it cannot be recovered. Furthermore, the Raman scattering cross section of the Au oxidation species is much smaller than that of 4-MBA. Due to both the small amount and small cross section of Au oxidation species, we did not observe Au-OH and Au-O signals in the present TERS spectra.

Regarding the issue of thiol desorption, the intensities of the benzene ring vibrational peaks at 1075 cm^{-1} did not decrease after illumination, indicating that the molecules hardly desorb from the surface. If the thiol molecules were oxidized to sulfonate species, the feature peak at around 600 cm^{-1} should be observed according to our previous work[13]. However, we didn't observe the feature peaks of sulfinate or sulfonate species, which indicates that the Au-S were not oxidized in the time scale of our experiments.

Supplementary Note 8

Spatial resolution of the TERS imaging system

To characterize the spatial resolution of the TERS system, TERS line scanning of a gold step edge on the Au(111) single crystal surface was carried out. As shown in Supplementary Figure 15, the step edge could produce a stronger TERS intensity than that at flat surface sites, resulting from the

stronger electromagnetic fields at the atomic step sites[14]. The spatial resolution is estimated to be better than 5 nm, which is in accordance with our previous results.

Supplementary Note 9

Estimation of the diffusion length OH·

The diffusion length of OH· can be estimated by $d = \sqrt{\tau D}$. where D is the diffusion coefficient of OH· in aqueous solution (2.8×10^{-9} m^2/s),[15] and τ is the lifetime of OH· in solution (10^{-9} s).[16, 17] Therefore, the diffusion length of OH· can be estimated to be about 1.7 nm.

Supplementary Note 10

Effect of the hot carrier transport after generation on the distribution of reaction products

The spatial distribution of the reaction is determined by the distribution of reactive hot carriers in real space, which is determined by the generation distribution of hot carriers and the transport of these carriers.[18, 19] The generation distribution is determined by the localized plasmonic electric field[18] (Supplementary Figure 17a). Following their generation, the subsequent transport of hot carriers will lead to the broadening of their distribution. The exponential attenuation model,[20-22] $N(x) = N_0\, e^{(-x/\iota)}$, can be used to estimate the distribution of hot carriers over a transport length x (Supplementary Figure 17b). Here, ι is the decay length of the reactive carriers. As shown in Supplementary Figure 17c, the carriers at a site x may transport from other sites $(x - \Delta x)$. The generation efficiency of the hot carriers at sites $(x - \Delta x)$ is $G(x - \Delta x)$. After the hot carriers move over a distance of Δx, their number arriving at site x is $G(x–\Delta x)$ $\mathrm{e}^{(-|\Delta x|/\iota)}$. The total number of hot carriers at site x can be given as:

$$D(x) = \sum_{\Delta x=-\infty}^{\infty} G(x - \Delta x)\ \mathrm{e}^{(-|\Delta x|/\iota)} = G(x) \otimes \mathrm{e}^{(-|x|/\iota)} \propto E(x)^2 \otimes \mathrm{e}^{(-|x|/\iota)} \qquad (1)$$

So the final distribution is the convolution of the generation distribution, which follows the distribution of the localized plasmonic electric field, with the exponential attenuation resulting from the carriers' transport.

Supplementary Figures

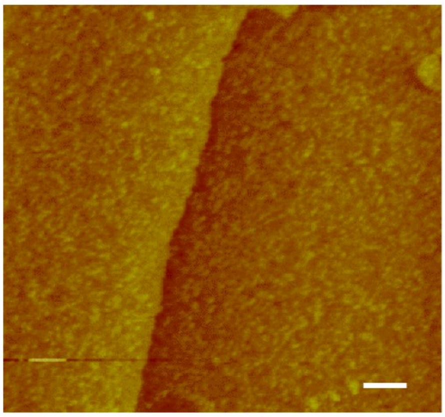

Supplementary Figure 1 | STM image of 4-MBA adsorbed Au(111) surface, the scale bar is 20 nm.

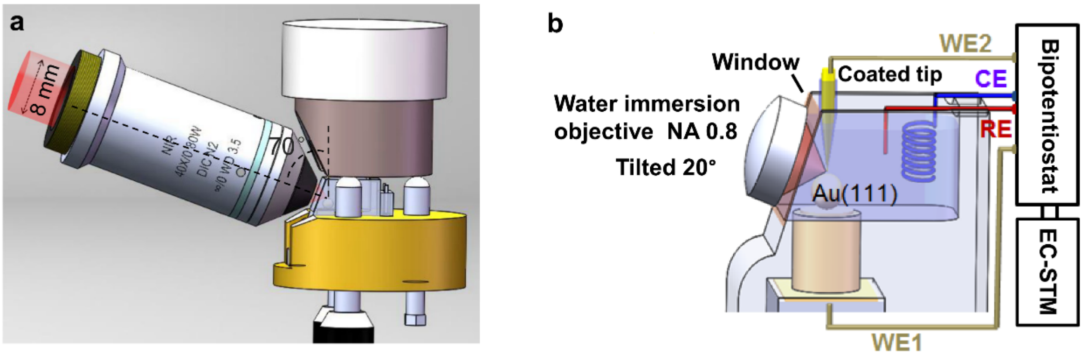

Supplementary Figure 2 | EC-TERS setup coupled with a water immersion objective for excitation and collection. **a** 3D drawing of the setup. The geometries of the STM scanner and base were modified to accommodate a water immersion objective with a short working distance and a high NA. **b** Schematic illustration of the spectroelectrochemical cell for EC-TERS.[23]

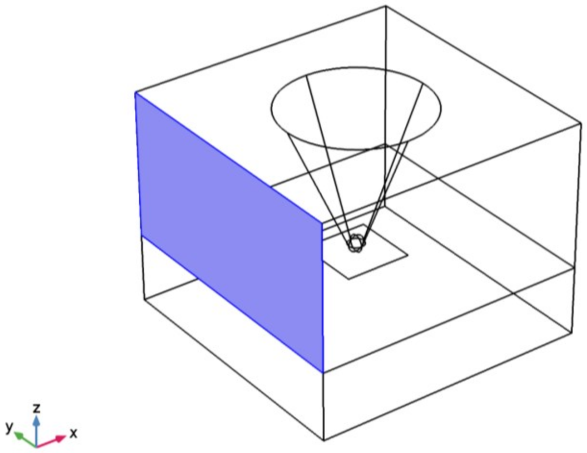

Supplementary Figure 3 | Model for COMSOL simulation of the electromagnetic field.

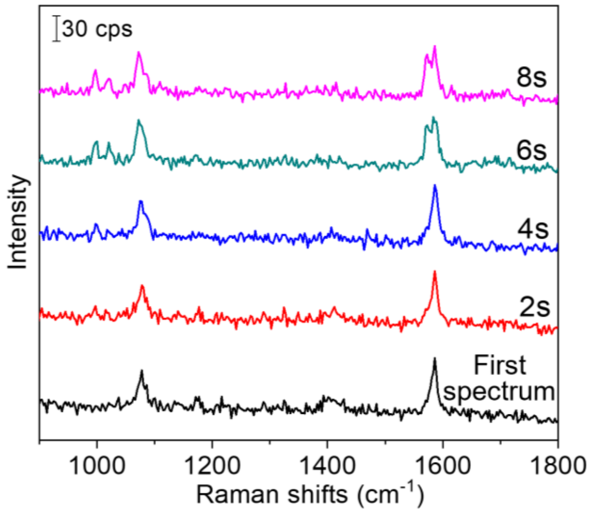

Supplementary Figure 4 | Control experiment to verify the role of laser illumination on the reaction. Before acquiring the TERS spectra, the laser light was switched off, and the Ag tip was approached to the 4-MBA adsorbed Au(111) and fixed for 1 min. The first spectrum acquired, while the laser was on, only showed the spectral feature of 4-MBA. The feature peaks of TP appeared gradually after continuous illumination on the tip-substrate gap for several seconds, demonstrating that laser illumination is required for the reaction. The potential of the substrate was set at the open circuit potential. The laser power was 1.1 mW.

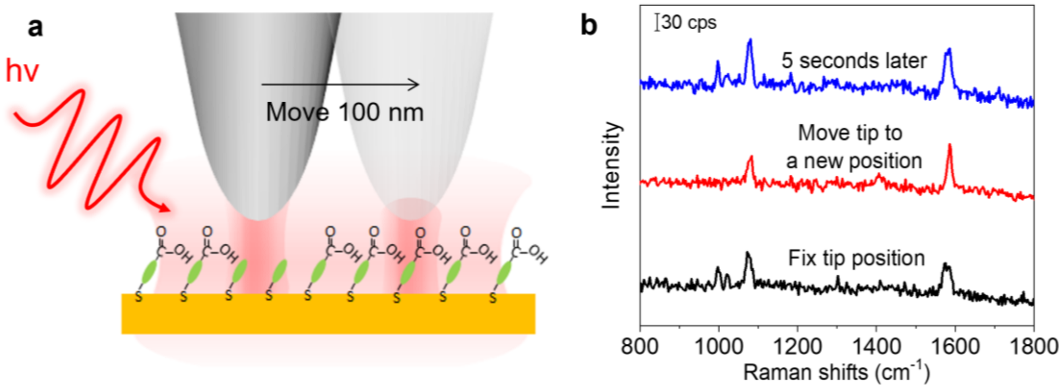

Supplementary Figure 5 | Control experiments to verify that the reaction was induced with the assistance of the plasmonic tip. **a** Schematic illustration of the control experiment. The size of the laser spot (r~0.5 µm) was much larger than the tip apex (r < 50 nm). After the reaction has been induced beneath the tip, the tip was moved 100 nm away to a new position in the laser spot. The TERS spectrum was acquired immediately after the movement. **b** The black spectrum was obtained after the tip has been fixed and illuminated for more than 5 s. The red spectrum was acquired on a new position in the laser spot immediately after the tip movement. The blue spectrum was collected after 5 s continuous exposure to laser illumination. The absence of the feature peaks of TP in the first spectrum (red spectrum) after tip movement indicates that the reaction could not be induced by the laser illumination alone in the absence of a plasmonic tip. The results indicate that the plasmonic tip is required to induce the reaction. The power was 1.1 mW.

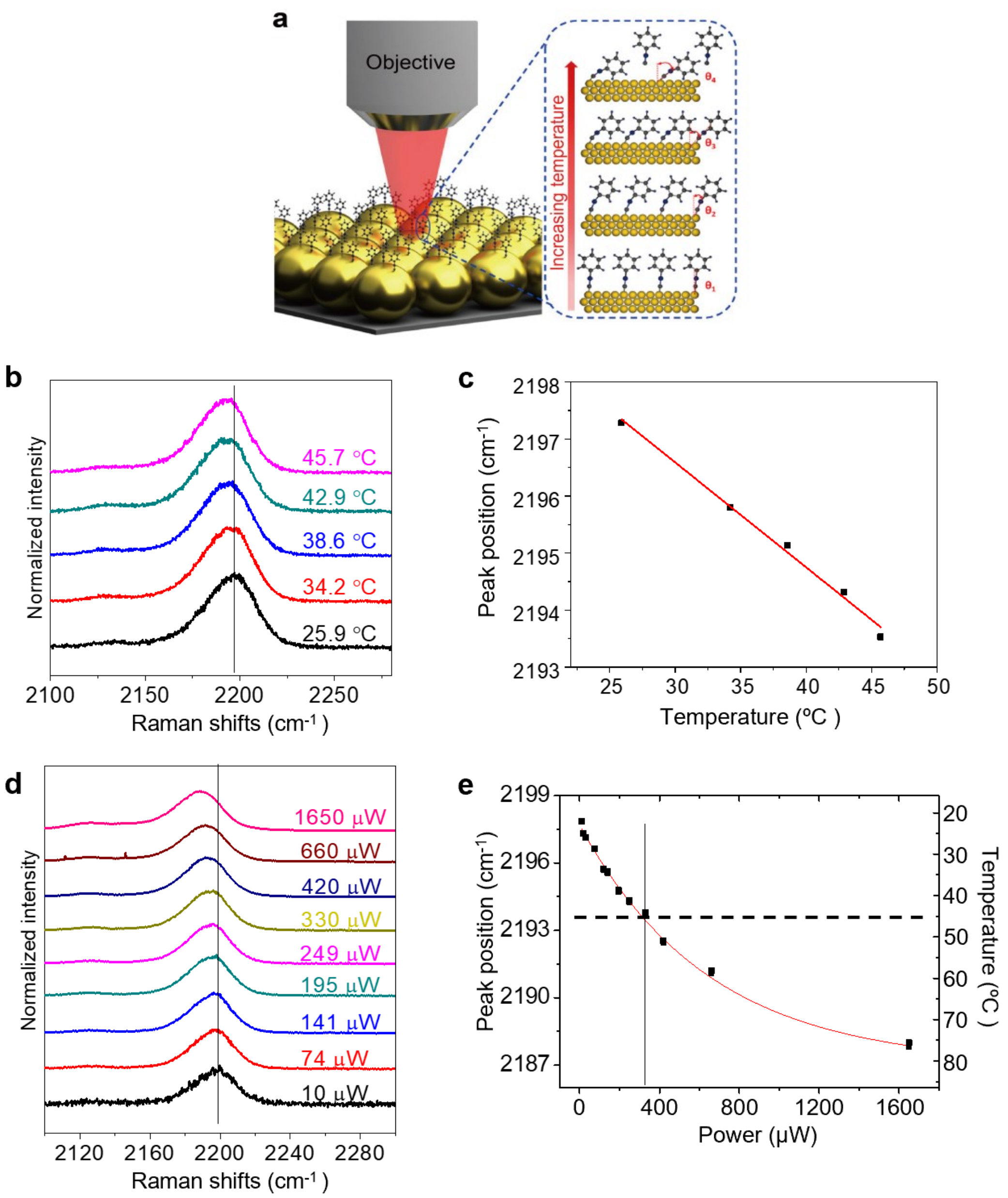

Supplementary Figure 6 | Measurement of the surface temperature on Au NPs heated by the plasmonic thermal effect. **a** Schematic illustration of the local temperature sensing using the phenyl isocyanide (PIC) probe molecules.[1] **b** Temperature-dependent SERS spectra of PIC in the spectral range of the N≡C stretching vibration. **c** Peak position of the N≡C peak as a function of temperature. The slope of the linear fitting curve is 0.184 cm^{-1}/ºC. **d** Power-dependent SERS spectra of the N≡C stretching vibration of PIC. **e** Position of the N≡C peak as a function of the laser power.

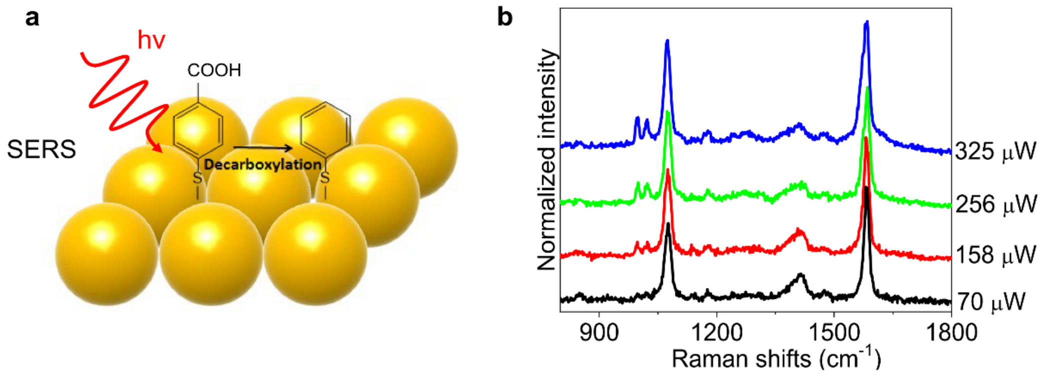

Supplementary Figure 7 | **a** Scheme of SERS study of the SP-induced decarboxylation on Au NPs. **b** Power-dependent SERS spectra of the 4-MBA adsorbed on Au NPs in $NaClO_4$ (pH 10). Each spectrum was acquired with an integration time of 2 s, following laser illumination on Au NPs substrate for 180 s. The spectra were acquired at a new position at different laser powers. The feature peaks of TP were observed in the laser power range of 158 ~325 μW.

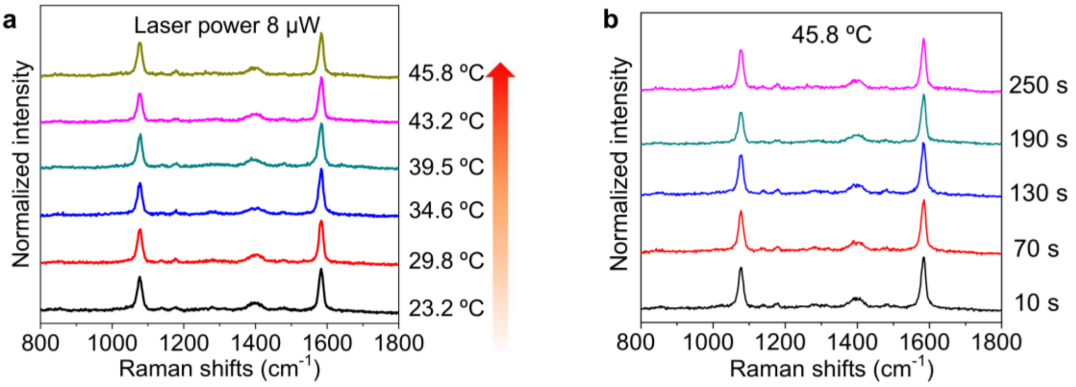

Supplementary Figure 8 | The thermal effect on the decarboxylated reaction of the 4-MBA molecules adsorbed Au NPs surface measured by SERS. **a** SERS spectra of 4-MBA under different heating temperatures. **b** A time series of SERS spectra of 4-MBA obtained under 45.8 ºC. The laser power was about 8 μW, and the acquisition time for each spectrum was 10 s. The feature peaks of TP were absent even when the Au NPs substrate was thermally heated to 45.8 ºC (the equivalent plasmonic heating temperature on Au NPs under illumination with 325 μW laser power).

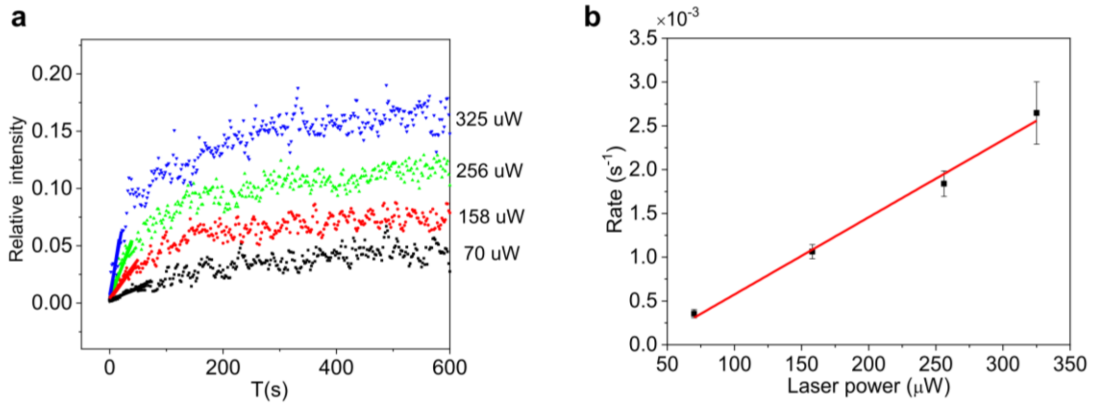

Supplementary Figure 9 | SERS study of the effect of the laser power on the reaction rate. **a** Relative area of the peak at 998 cm^{-1} (feature peak of TP) to that at 1075 cm^{-1} (contributed from both TP and 4-MBA) as a function of time. **b** Dependence of the reaction rate on the laser power. The reaction rate was obtained from the slope of the initial linear part of the time dependent intensity plots. The error bars represent the standard deviations for three measurements.

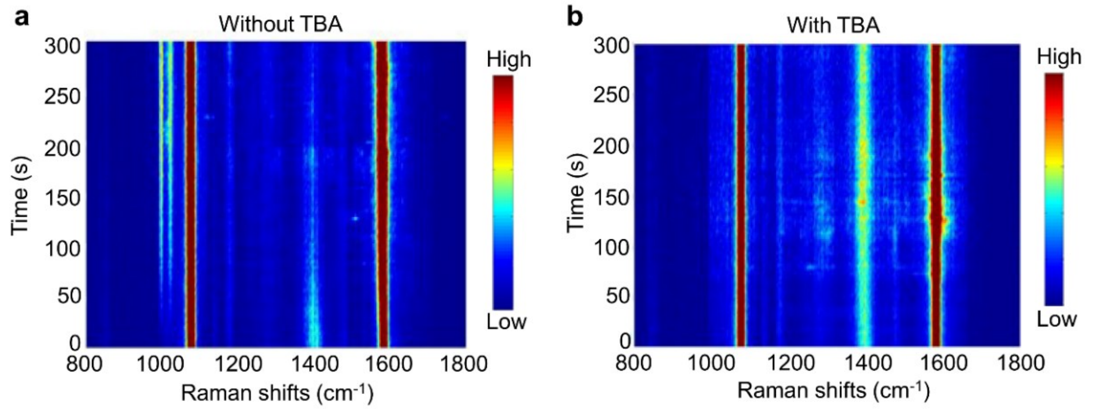

Supplementary Figure 10 | Color-coded plot of the time series SERS spectra of 4-MBA in $NaClO_4$ (pH 10) **a** without and **b** with 2.7 M TBA. The laser power was 0.3 mW.

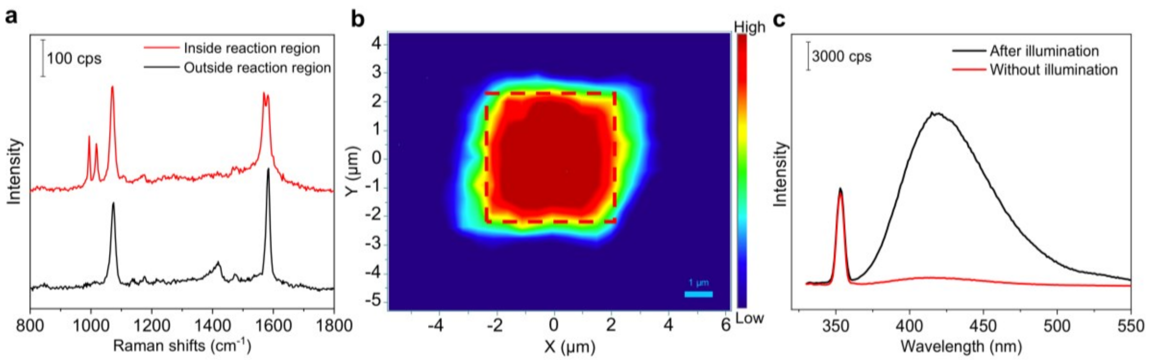

Supplementary Figure 11 | Ex-situ fluorescence and SERS experiments to verify the generation of OH· during surface plasmon induced decarboxylation of 4-MBA. **a** SERS spectra inside (red) and outside (black) the reaction region. **b** SERS imaging of the induced decarboxylation reaction region using $\frac{I_{998\ cm^{-1}}}{I_{1075\ cm^{-1}}}$. Before the imaging, the laser with a power of 0.33 mW was scanned over a region of about 4.5 μm × 4.5 μm for about 3.5 h to induce the decarboxylation. The solution contained 3 mM TA and 0.1 M $NaClO_4$ (pH 10). The potential was controlled at –0.4 V referring to Pt quasi-reference electrode. **c** Fluorescence emission spectra recorded from the solution after the SERS experiment. The fluorescent peak at around 425 nm is from the emission of 2-hydroxyterephthalic acid, which is the reaction product between OH· and TA. The emission spectra were excited by 315 nm monochromatic light.

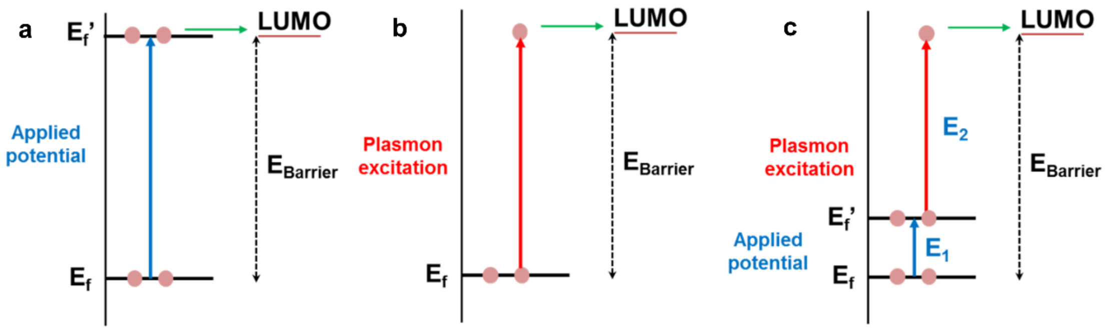

Supplementary Figure 12 | Scheme of the modulation of the reaction by electrochemical potential and plasmon excitation. The surface reaction can be driven by the applied potential **a** and plasmon excitation **b** alone. **c** The energy of electrons can be synergistically tuned by the applied potential (E_1) and the plasmon excitation (E_2) to overcome the energy barrier. In our work, E_2 was fixed and E_1 was modulated to change the reaction rate. The reference investigated the variation of E_1 needed to initiate the polymerization (means under a fixed reaction rate) while E_2 changed[24].

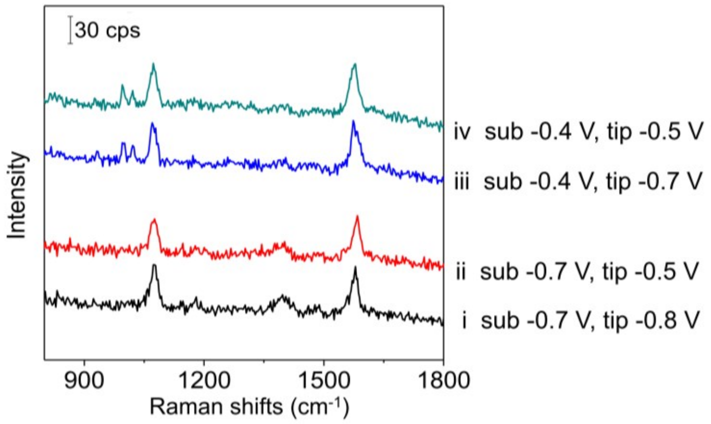

Supplementary Figure 13 | TERS spectra obtained at different tip potentials and substrate potentials as indicated in the figure. All spectra were acquired with an integration time of 2 s after laser illumination on the plasmonic tip-sample gap for 30 s. The laser power was 0.7 mW. Since the tip-substrate distance is proportional to ln ($V_{bias}/I_{tunnelling}$)， the tunneling current was set proportional to the bias to keep the distance constant and avoid the effect of distance change on the plasmonic coupling while changing the potential.

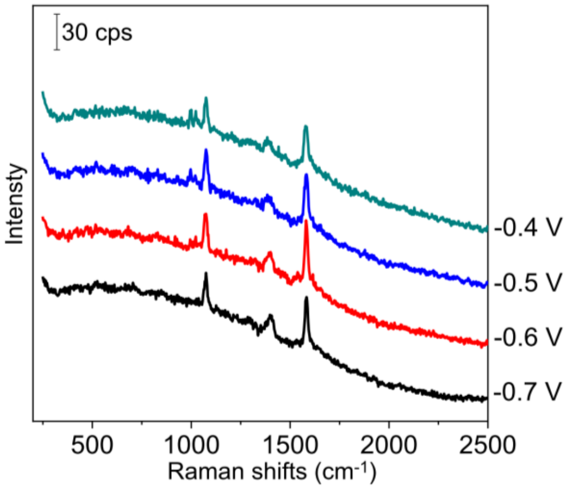

Supplementary Figure 14 | EC-TERS spectra before background subtraction. The spectra were offset for clarity. The backgrounds of the original TERS spectra do not show an obvious shift with the potential change. The TERS background is mainly originated from the photoluminescence of metals, which is shaped by the SPR scattering in the gap.[25] Therefore, from the TERS background we can get a clue of the gap SPR. The backgrounds of the TERS spectra do not shift with the change of the potential, indicating that the potential did not lead to an obvious change in the SPR response.

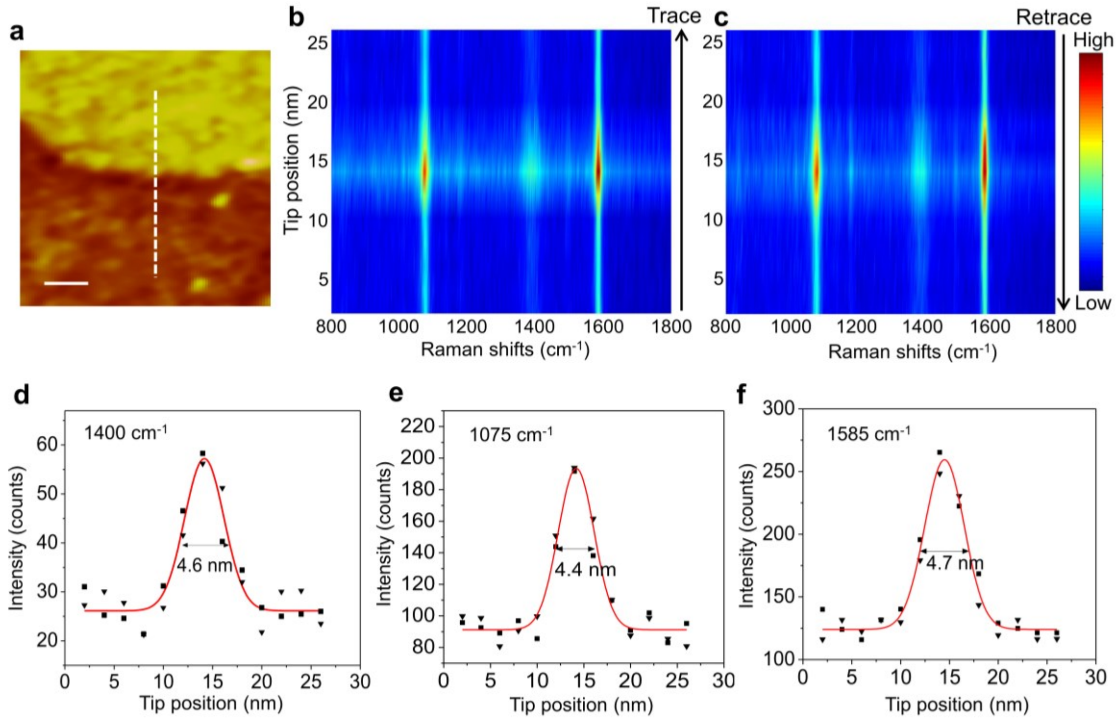

Supplementary Figure 15 | TERS line scanning across an atomic step on the single crystal surface to charaterize the spatial resolution of the TERS imaging system. **a** STM image of the Au(111) suface with adsorbed 4-MBA obtained in solution with a Ag tip, the scale bar is 6 nm. **b**, **c** Trace and retrace TERS line mapping of an atomic step along the dashed line in **a**. **d-f** Plots of intensities of the TERS peaks (1400 cm^{-1}, 1075 cm^{-1}, and 1585 cm^{-1}) with the tip position.

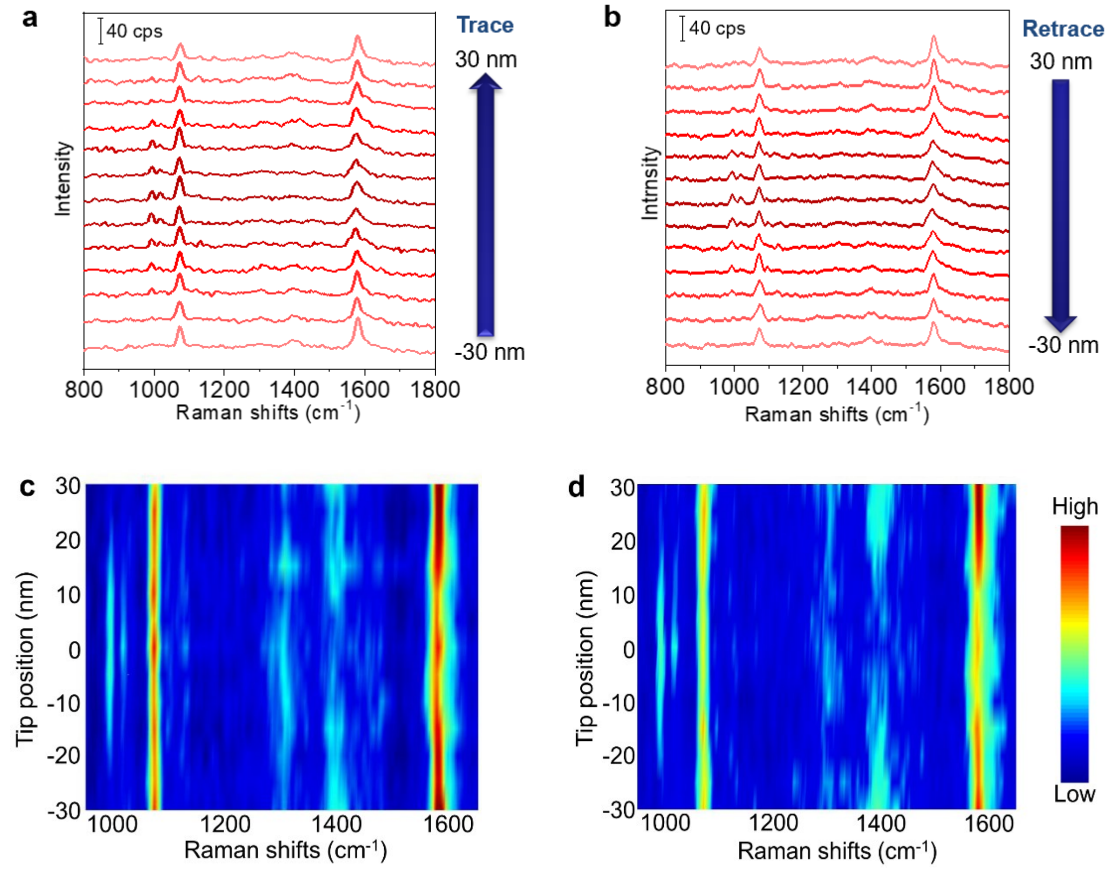

Supplementary Figure 16 | Line-trace and retrace TERS investigation across the center of the reaction region (induced at a substrate potential of –0.4 V) in the tip-substrate gap. **a**, **b** Line-trace and retrace TERS spectra obtained across the reaction region. **c**, **d** Color-coded intensity map of line scan TERS images. The trace image **c** is also shown in Figure 4a in the main text. The profile of the reaction region is shown in Figure 4b. The trace and retrace images show a good reproducibility.

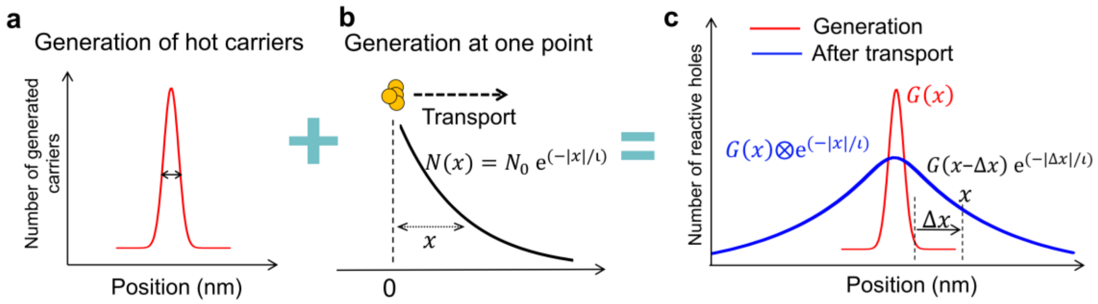

Supplementary Figure 17 | Schematic illustration of the hot carrier generation and transport. **a** Distribution of the generated hot carriers, which follows the distribution of the localizd plasmonic electric field. **b** Transport of the hot carriers after generation. **c** Overall distribution of hot carriers, which is a convolution of carrier generation and transport.

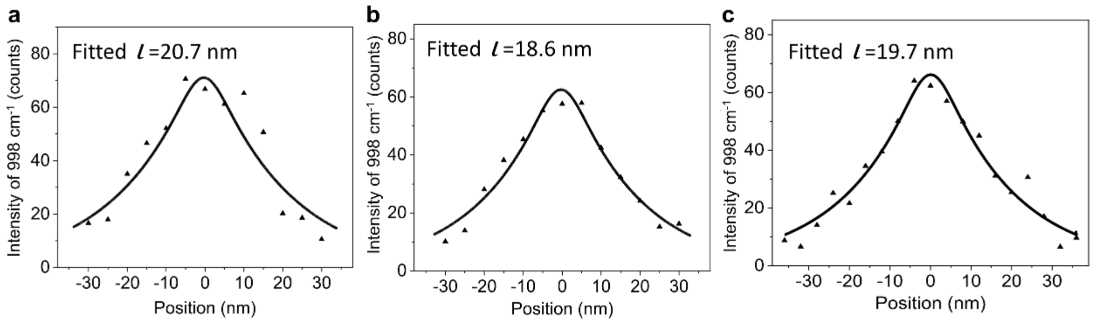

Supplementary Figure 18 | **a-c** are three TERS traces of the reaction region induced at –0.4 V. The fitted lines were obtained by using the function $AE(x)^2 \otimes e^{(-|x|/\iota)} \otimes R(x)$, where A is a constant, $E(x)^2$ is the profile of plasmonic electric field intensity in Fig. 4d in the manuscript, $R(x)$ is the TERS spatial resolution profile, obtained from Supplementary Fig. 14. The fitted values of transport distance (ι) of reactive hot carriers obtained from three traces are 20.7 nm, 18.6 nm and 19.7 nm. The standard deviation is about 1.1 nm.

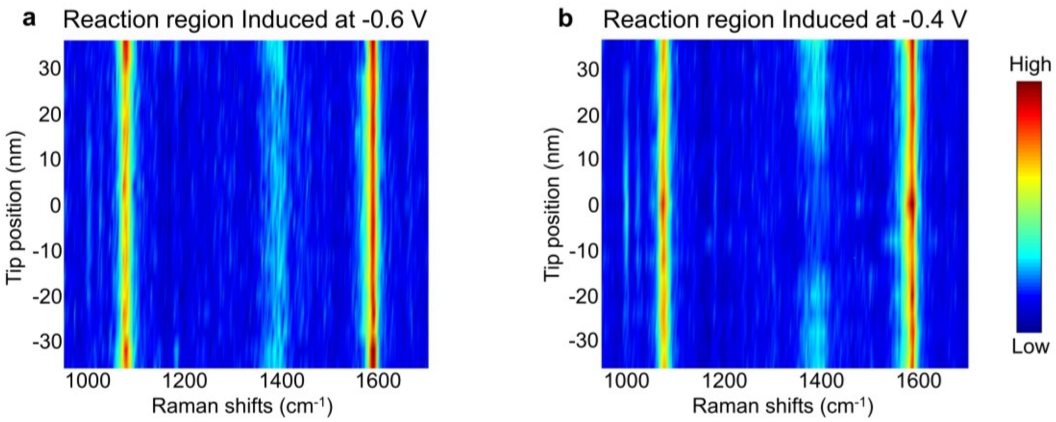

Supplementary Figure 19 | **a**, **b** Color-coded TERS intensity map across reaction regions induced on the substrate at potentials of –0.6 V and –0.4 V, respectively. The laser power used was 0.7 mW. The background was subtracted for a better view of the change in Raman intensity. The profile of the reaction region is shown in Figure 4f.

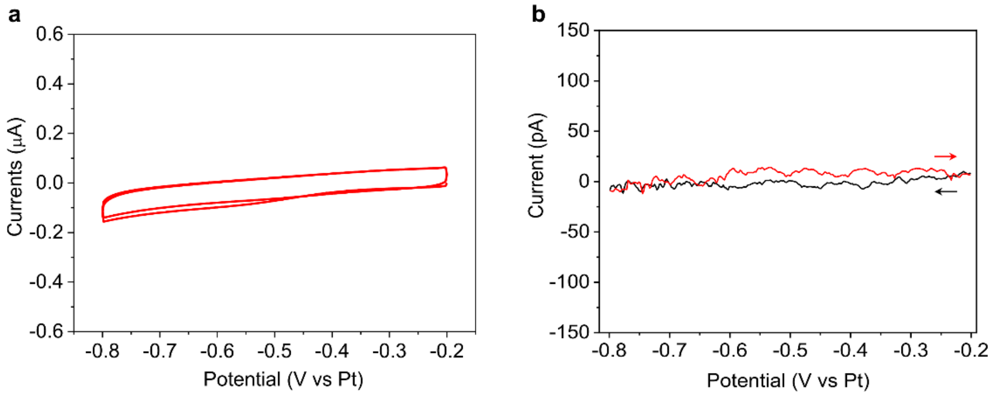

Supplementary Figure 20 | **a** CV of the 4-MBA/Au(111) in 0.1 M $NaClO_4$ (pH 10). The potential window covers the studied potential range in EC-TERS, scan rate: 50 mV/s. The charge current of the double layer and a weak cathodic peak attributed to the oxygen reduction at about –0.5 V was observed in the applied potential window. **b** CV of the insulated Ag tip in 0.1 M $NaClO_4$ (pH 10), scan rate: 50 mV/s. The insulation could effectively decrease exposure area of the tip, as a result, the electrochemical currents including the charge current and Faradic current could be decreased to less than 50 pA, which could minimize their interference with the tunneling current.

Supplementary Tables

Supplementary Table 1 Peak assignments of 4-MBA adsorbed on Au(111)

Peak position (cm^{-1})	Assignment
1075	Benzene ring ν_{12} vibration[26, 27]
1185	C-H bending vibration δ_{CH}[28, 29]
1400	Stretching vibration of carboxylic group[26, 27]
1585	Benzene ring ν_{8a} vibration[26, 27]

Supplementary Table 2 Peak assignments of thiophenol (TP) adsorbed on Au(111)

Peak position (cm^{-1})	Assignment
998	Benzene ring in-plane deformation mode r-i-d + C-C stretching mode ν_{CC}[30]
1020	C-C stretching mode ν_{CC} +C-H deformation mode δ_{CH}[30]
1075	Benzene ring ν_{12} vibration
1577	Benzene ring ν_{8a} vibration

Supplementary References

1. Hu, S. et al. Quantifying surface temperature of thermoplasmonic nanostructures. *J. Am. Chem. Soc.* **140**, 13680-13686 (2018).
2. Kale, M.J., Avanesian, T. & Christopher, P. Direct photocatalysis by plasmonic nanostructures. *ACS Catal.* **4**, 116-128 (2014).
3. Linic, S., Aslam, U., Boerigter, C. & Morabito, M. Photochemical transformations on plasmonic metal nanoparticles. *Nat. Mater.* **14**, 567-576 (2015).
4. Govorov, A.O. & Richardson, H.H. Generating heat with metal nanoparticles. *Nano Today* **2**, 30-38 (2007).
5. Christopher, P., Xin, H. & Linic, S. Visible-light-enhanced catalytic oxidation reactions on plasmonic silver nanostructures. *Nat. Chem.* **3**, 467-472 (2011).
6. Mukherjee, S. et al. Hot electrons do the impossible: plasmon-induced dissociation of H_2 on Au. *Nano Lett.* **13**, 240-247 (2013).
7. Kim, K.H., Watanabe, K., Mulugeta, D., Freund, H.-J. & Menzel, D. Enhanced photoinduced desorption from metal nanoparticles by photoexcitation of confined hot electrons using femtosecond laser pulses. *Phys. Rev. Lett.* **107**, 047401 (2011).
8. Christopher, P., Xin, H., Marimuthu, A. & Linic, S. Singular characteristics and unique chemical bond activation mechanisms of photocatalytic reactions on plasmonic nanostructures. *Nat. Mater.* **11**, 1044-1050 (2012).
9. Swearer, D.F. et al. Heterometallic antenna–reactor complexes for photocatalysis. *Proc. Natl. Acad. Sci.* **113**, 8916-8920 (2016).
10. Ishibashi, K., Fujishima, A., Watanabe, T. & Hashimoto, K. Quantum yields of active oxidative species formed on TiO_2 photocatalyst. *J. Photoch. Photobio. A* **134**, 139-142 (2000).
11. Chen, X., Zhang, J., Fu, X., Antonietti, M. & Wang, X. Fe-g-C_3N_4-catalyzed oxidation of benzene to phenol using hydrogen peroxide and visible light. *J. Am. Chem. Soc.* **131**, 11658-11659 (2009).
12. Nosaka, Y. & Nosaka, A.Y. Generation and detection of reactive oxygen species in photocatalysis. *Chem. Rev.* **117**, 11302-11336 (2017).

13. Su, H.-S. et al. Probing the local generation and diffusion of active oxygen species on a Pd/Au bimetallic surface by tip-enhanced Raman spectroscopy. *J. Am. Chem. Soc.* **142**, 1341-1347 (2020).
14. Zhong, J.-H. et al. Probing the electronic and catalytic properties of a bimetallic surface with 3 nm resolution. *Nat. Nanotechnol.* **12**, 132 (2017).
15. Schwarz, H.A. Applications of the spur diffusion model to the radiation chemistry of aqueous solutions. *J. Phys. Chem.* **73**, 1928-1937 (1969).
16. Pryor, W.A. Oxy-radicals and related species: their formation, lifetimes, and reactions. *Annu Rev Physiol* **48**, 657-667 (1986).
17. Xiang, Q., Yu, J. & Wong, P.K. Quantitative characterization of hydroxyl radicals produced by various photocatalysts. *J. Colloid Interf. Sci.* **357**, 163-167 (2011).
18. Narang, P., Sundararaman, R. & Atwater, H.A. Plasmonic hot carrier dynamics in solid-state and chemical systems for energy conversion. *Nanophotonics* **5**, 96-111 (2016).
19. Cortés, E. et al. Plasmonic hot electron transport drives nano-localized chemistry. *Nat. Commun.* **8**, 14880 (2017).
20. Sze, S.M., Moll, J.L. & Sugano, T. Range-energy relation of hot electrons in gold. *Solid State Electron.* **7**, 509-523 (1964).
21. Soshea, R.W. & Lucas, R.C. Attenuation length of hot electrons in gold. *Phys. Rev.* **138**, A1182-A1188 (1965).
22. Stollenwerk, A.J. et al. Effect of interface band structure on hot-electron attenuation lengths in Au thin films. *Phys. Rev. B* **77**, 033416 (2008).
23. Huang, S.-C. et al. Electrochemical tip-enhanced Raman spectroscopy with improved sensitivity enabled by a water immersion objective. *Anal. Chem.* **91**, 11092-11097 (2019).
24. Pensa, E. et al. Spectral screening of the energy of hot holes over a particle plasmon resonance. *Nano Lett.* **19**, 1867-1874 (2019).
25. Lin, K.-Q. et al. Plasmonic photoluminescence for recovering native chemical information from surface-enhanced Raman scattering. *Nat. Commun.* **8**, 14891 (2017).
26. Michota, A. & Bukowska, J. Surface-enhanced Raman scattering (SERS) of 4-mercaptobenzoic acid on silver and gold substrates. *J. Raman Spectrosc.* **34**, 21-25

(2003).

27. Olson, T.Y. et al. Hollow gold–silver double-shell nanospheres: structure, optical absorption, and surface-enhanced Raman scattering. *J. Phys. Chem. C* **112**, 6319-6329 (2008).

28. Orendorff, C.J., Gole, A., Sau, T.K. & Murphy, C.J. Surface-enhanced Raman spectroscopy of self-assembled monolayers: sandwich architecture and nanoparticle shape dependence. *Anal. Chem.* **77**, 3261-3266 (2005).

29. Kudelski, A. Surface-enhanced Raman scattering study of monolayers formed from mixtures of 4–mercaptobenzoic acid and various aromatic mercapto-derivative bases. *J. Raman Spectrosc.* **40**, 2037-2043 (2009).

30. Blum, C. et al. Tip-enhanced Raman spectroscopy – an interlaboratory reproducibility and comparison study. *J. Raman Spectrosc.* **45**, 22-31 (2014).
